# Socio-demographic characteristics, diet and health among food insecure UK adults: cross-sectional analysis of the International Food Policy Study

**DOI:** 10.1017/S1368980020000087

**Published:** 2020-10

**Authors:** Amy Yau, Martin White, David Hammond, Christine White, Jean Adams

**Affiliations:** 1 Centre for Diet and Activity Research, MRC Epidemiology Unit, University of Cambridge, Cambridge, UK; 2 School of Public Health and Health Systems, University of Waterloo, Waterloo, Canada

**Keywords:** Food insecurity, Socio-demographic characteristics, Stress, Diet, Health outcomes, Overweight

## Abstract

**Objective:**

To estimate food insecurity (FI) prevalence among UK adults and investigate associations with socio-demographic characteristics, diet and health.

**Design:**

Weighted cross-sectional survey data. FI was measured using the USDA Adult Food Security Survey Module. Data were analysed using adjusted logistic regression models.

**Setting:**

United Kingdom.

**Participants:**

2551 participants (aged 18–64 years); sub-sample (*n* 1949) used to investigate association between FI and overweight.

**Results:**

FI prevalence was 24·3 %. Higher odds of FI were observed among participants who reported that making ends meet was difficult *v*. easy (OR 19·76, 95 % CI 13·78, 28·34), were full-time students *v*. non-students (OR 3·23, 95 % CI 2·01, 5·18), had low *v*. high education (OR 2·30, 95 % CI 1·66, 3·17), were male *v*. female (OR 1·36, 95 % CI 1·01, 1·83) and reported their ethnicity as mixed (OR 2·32, 95 % CI 1·02, 5·27) and white other (OR 2·04, 95 % CI 1·04, 3·99) *v*. white British. Odds of FI were higher in participants living with children *v*. alone, especially in single-parent households (OR 2·10, 95 % CI 1·19, 3·70). Odds of FI decreased per year of increase in age (OR 0·95, 95 % CI 0·94, 0·96) and were lower in participants not looking for work *v*. full-time employed (OR 0·60, 95 % CI 0·42, 0·87). Food insecure *v*. food secure adults had lower odds of consuming fruits (OR 0·59, 95 % CI 0·47, 0·74) and vegetables (OR 0·68, 95 % CI 0·54, 0·86) above the median frequency, and higher odds for fruit juice (OR 1·39, 95 % CI 1·10, 1·75). Food insecure *v*. food secure adults had higher odds of reporting unhealthy diets (OR 1·65, 95 % CI 1·31, 2·10), poor general health, (OR 1·90, 95 % CI 1·50, 2·41), poor mental health (OR 2·10, 95 % CI 1·64, 2·69), high stress (OR 3·15, 95 % CI 2·42, 4·11) and overweight (OR 1·32, 95 % CI 1·00, 1·75).

**Conclusions:**

FI prevalence was high and varied by socio-demographic characteristics. FI was associated with poorer diet and health.

Food security is ‘when all people at all times have physical, social and economic access to sufficient, safe and nutritious food which meets their dietary needs and food preferences for an active and healthy life^(1)^.’ Despite being a high-income country, prevalence of individual-level food insecurity (FI) was estimated at 8 % among adults^(2)^ and over 20 % in low-income households, in the UK in 2016^(3)^. In 2018/2019, the Trussell Trust (the UK’s largest network of food banks) provided emergency food aid to 1 006 050 adults, five times more than in 2012/2013^(4)^.

The cost of living has increased in the UK since the mid-2000s, whilst wages have stagnated^(5)^. For example, the cost of domestic fuel and transportation increased approximately 45 and 81 % in the last decade, respectively^(6)^. Due to welfare reform and austerity measures in the UK, individuals receiving benefit payments have experienced cuts and delays to their payments^(7–9)^. Increasing childcare costs is further cited as an increasingly large financial burden on families^(6)^. Food prices have also increased during this time^(6)^. Consequently, individuals with low incomes may face an absolute shortage of food or a shortage of healthier foods due to their high cost relative to less healthy foods^(10)^. Indeed, lower-income households in the UK spend a larger proportion of their total expenditure on food (17 %) compared to higher-income households (8 %)^(11)^. Lower-income households also spend a larger proportion of their food budget on basic necessities, such as bread and milk, and a smaller proportion on vegetables compared to higher-income households^(11,12)^.

FI has been reported in the academic literature since the 1990s^(13)^ and has been found to be associated with poor diet and health. In a systematic review, food insecure adults were found to have lower intake of fruit, vegetables and dairy compared to food secure adults^(14)^. Increased rates of mental health problems, diabetes, hypertension and hyperlipidaemia among food insecure adults, compared to food secure adults, have also been reported^(15)^. Findings on the association between FI and obesity have been mixed. However, a positive association between FI and obesity is more consistently reported among women than in men^(16)^, suggesting that the association could differ between population subgroups. In Canada, FI prevalence was reported to be higher among Aboriginal adults and individuals without a degree, as well as in households that relied on social assistance, or had children^(17)^. Despite this wealth of evidence, it is almost exclusively based on data from North America. Findings from North America may not be generalisable to other contexts due to differences in economic situation and food environment context (including food prices, food culture and food accessibility)^(5,18)^.

In the UK, associations between FI and age and ethnicity have been reported in women living in the city of Bradford^(19)^. FI was found to be associated with the presence of common mental disorders and poorer health among mothers in the Born in Bradford cohort^(20,21)^. Single-parent households and households with more children have also been reported to have increasingly higher risk of FI compared to other household types^(22)^. Some UK studies have examined associations with FI using food bank usage as a proxy measure of FI. Food banks provide emergency food parcels to alleviate hunger^(23)^. However, food bank usage may be an inaccurate measure of FI. Food banks are not the only source of food aid, and their use is stigmatised^(24)^. Thus, food bank usage is likely to underestimate the prevalence of FI^(25)^. Further, food bank users have been found to experience more financial strain and adverse life events, compared to other disadvantaged groups in which FI is prevalent^(26)^, meaning that users may not be representative of all those experiencing FI.

Few studies have investigated the prevalence of FI, variations within the population and associations with diet and health in the general UK population. In this study, we aimed to estimate prevalence of FI among UK adults using a national sample of the general population and investigate associations between FI and socio-demographic characteristics (sex, age, ethnicity, household composition, employment status, student status, ability to make ends meet and education), diet (fruit and vegetable intake frequency and self-rated healthiness of diet) and health (self-rated general health, mental health and stress, and BMI).

## Methods

### Study population

We used cross-sectional UK data from wave 1 of the International Food Policy Study (IFPS)^(27)^. Participants were recruited through the online *Nielsen Consumer Insights Global Panel* and partner panels, which select panel members using both probability and non-probability sampling methods. Email invitations with unique survey access links were sent to a random sample of panellists within a specified age range; panellists known to be ineligible were not invited. To account for differential response rates by age, approximately 2000 participants aged 18–30 years and 2000 participants aged 31–64 years were recruited. In total, 4047 UK adults were recruited for the baseline survey conducted in December 2017. Full details regarding the IFPS methods can be found elsewhere^(27)^. In our analysis, participants were excluded for incomplete adult food security status (*n* 767) and missing diet and health outcome data (*n* 729). This resulted in an analytical sample of 2551 participants. Due to a large number of missing BMI values (*n* 602), we used a smaller analytical sub-sample (*n* 1949) to explore the association between adult food security and BMI.

### Measuring adult food security

Adult food security was measured using the validated Adult Food Security Survey Module (AFSSM) developed by the United States Department of Agriculture, which is the adult portion of the most commonly used measure of FI globally (the Household Food Security Survey Module – HFSSM)^(28)^. Minor changes in wording were made for the IFPS to adapt the measure for use in an online self-administered survey. The AFSSM comprises ten questions related to household food sufficiency in the last 12 months, with a total potential score of 0–10. Participants receive one point for each affirmative response (‘yes’, ‘often’, ‘sometimes’, ‘almost every month’ or ‘some months but not every month’) given. Questions were related to having enough to eat, worrying about food, balanced meals, reducing sizes of meals or skipping meals, hunger and weight loss (see online Supplementary Table S1). Questions were administered in a three-stage design, reducing participant burden, as participants could potentially be confirmed as food secure using the first three questions. Further questions were only then asked if these questions highlighted potential FI. AFSSM assigns participants to four categories: high food security (score 0), marginal food security (score 1–2), low food security (score 3–5) and very low food security (score 6–10). For our analysis, we categorised participants as: food secure (score 0–2) or food insecure (score 3–10). The majority of participants who were excluded for incomplete adult food security status (*n* 599) had missing values due to a systematic programming error that prevented some eligible participants from progressing into the second stage.

### Correlates

We used self-reported data available from the IFPS questionnaire that related to socio-demographic characteristics, diet and health to explore associations with FI.

#### Socio-demographic characteristics

Participants reported their sex (male and female), age (continuous), ethnicity (white British, white other, mixed, Asian, black and other/unknown), employment status (full-time employment, part-time employment, looking for work and not looking for work), student status (full-time, part-time and not studying) and ability to make ends meet (difficult, neither easy nor difficult and easy). Participants also reported the highest level of education completed, which we categorised as: low (GCSE or below – school leaving qualifications taken at around age 16 years), medium (A level and NVQ level 4–5 – school leaving qualifications taken at around age 18 years) and high (degree or equivalent). Participants reported their current living situation, which we used to categorise participants’ household composition as living with: no other adults and no children (i.e. alone), other adults and no children, no other adults and with children (i.e. single-parent household), and other adults and children.

#### Frequency of fruit and vegetable intake

In lieu of more detailed dietary assessment, participants were asked how many times they consumed fruits, vegetables (including lettuce salads but excluding all types of potatoes) and fruit juice, using questions adapted from the validated 2017 Behavioural Risk Factor Surveillance System fruit and vegetable intake module, which was developed in the Unite States.^(29–31)^ Participants provided answers per day, week, month or year, as preferred, which we then converted to the standard indicator of frequency per day. To address outliers, intake frequency was capped at the mean plus three standard deviations (stratified by sex) and higher values were reassigned the cap value, as recommended by *Pérez* 2002^(32)^. For vegetables, we first excluded two values (634 and 1·03*e*^13^) due to implausibility before calculating the cap value.

#### Self-rated healthiness of diet and health

Participants rated the healthiness of their diet, their general health and their mental health as: poor, fair, good, very good or excellent. We categorised responses as: poor (poor and fair) or good (good, very good and excellent). Participants were also asked about the amount of stress in their lives and reported whether most days were: not at all stressful, not very stressful, a bit stressful, very stressful or extremely stressful. We categorised answers as: low stress (not at all stressful, not very stressful and a bit stressful) or high stress (very stressful and extremely stressful).

#### BMI

We calculated BMI (weight/height^2^) for 1949 participants in the analytical sub-sample from self-reported height and weight, categorising participants as: not overweight (BMI ≤ 25) or overweight (BMI > 25). Other participants had missing height and/or weight values (*n* 511) or were excluded due to an extreme BMI value (<14 or >48), extreme height (<3 ft/0·91 m or >7 ft/2·13 m) and/or extreme weight (<45 lb/20·4 kg or >1100 lb/499·0 kg). The large number of missing and implausible weight values was partly due to a programming error, which meant participants were not able to answer using British Imperial measures (stones and pounds), commonly used units of body weight in the UK.

### Statistical methods

Wald tests were used to test differences between food secure and food insecure adults in all measured correlates. Adjusted logistic regression models were used to estimate odds, with 95 % CI, of FI across socio-demographic subgroups (sex, age, ethnicity, household composition, student status, employment status, ability to make ends meet, education), mutually adjusting for other socio-demographic characteristics. Adjusted logistic regression models were also used to estimate odds, with 95 % CI, of food insecure adults consuming above the median intake frequency for fruit, vegetables and fruit juice, and reporting poor healthiness of diet, general health, mental health, high stress and overweight, compared to food secure adults, adjusting for sex, age, ethnicity and household composition. Interaction between sex, age, ethnicity, and household composition and adult food security on their effect on diet and health was tested (see online Supplementary Table S2). Where interaction terms were statistically significant, stratified results are presented. We report significant interactions with age (continuous) by age groups: 18–24, 25–30, 31–39, 40–49, 50–59, and 60–64 years.

Weighted data were used in all analyses. Post-stratification sample survey weights were based on 2016 mid-year estimates and adjusted the study sample to be representative of the UK adult population in terms of sex, age and region of residence (see online Supplementary Table S3). Sample weights were scaled separately for the main analytic sample and the BMI sub-sample. Significance levels were set at a two-tailed *P*-value ≤ 0·05 for all tests. All analyses were performed using Stata/SE 13.

### Sensitivity analyses

We present two adjusted logistic regression models for the association between socio-demographic characteristics and FI. Model 1 is adjusted for sex, age, ethnicity and household composition. Model 2 is additionally adjusted for markers of socio-economic position: employment status, student status, ability to make ends meet and education. In our main analyses for associations between FI and diet and health, we did not adjust our logistic regression models for markers of socio-economic position, which we theorised to be determinants of FI rather than confounders of any relationships with diet and health. In our sensitivity analyses, we tested this assumption by additionally adjusting these models for employment status, student status, ability to make ends meet and education (see Table 1 for distribution of characteristics). The association between sex, age, ethnicity, and household composition, and diet and health outcomes are presented in online Supplementary Table S4.

**Table 1:** Weighted distribution of socio-demographic characteristics among full analytic sample (*n* 2551) and BMI sub-sample (*n* 1949)

	Full analytical sample								BMI sub-sample							
	Overall		Food secure		Food insecure				Overall		Food secure		Food insecure			
	%	95 % CI	%	95 % CI	%	95 % CI	Pearson’s *F* statistic	*P*-value	%	95 % CI	%	95 % CI	%	95 % CI	Pearson’s *F* statistic	*P*-value
Total			75·7	73·7, 77·6	24·3	22·4, 26·3	N/A				78·2	76·0, 80·3	21·8	19·7, 24·1	N/A	
Sex																
Male	48·9	46·7, 51·2	48·3	45·7, 50·9	50·9	46·2, 55·6	0·88	0·35	51·3	48·7, 53·9	50·4	47·5, 53·4	54·4	48·7, 59·9	1·45	0·23
Female	51·1	48·8, 53·3	51·7	49·1, 54·3	49·1	44·4, 53·8			48·7	46·1, 51·3	49·6	46·7, 52·5	45·7	40·1, 51·3		
Age																
Median		44		46		36	113·94	<0·0001		45		47		37	76·16	<0·0001
IQR		32, 54		34, 56		28, 46				32, 55		34, 56		28, 46		
Ethnicity																
White British	85·2	83·5, 86·7	86·5	84·6, 88·2	81·1	77·1, 84·6	3·25	0·01	84·2	82·2, 86·0	85·3	83·0, 87·3	80·4	75·7, 84·4	3·10	0·01
White other	4·6	3·8, 5·7	4·4	3·5, 5·5	5·3	3·3, 8·5			5·2	4·2, 6·4	5·3	4·2, 6·7	4·8	2·9, 7·8		
Mixed	2·5	1·9, 3·3	1·8	1·2, 2·6	4·8	3·3, 7·1			2·8	2·1, 3·8	1·9	1·3, 3·0	5·9	3·7, 9·1		
Asian	4·1	3·3, 5·1	3·8	2·9, 4·9	5·1	3·4, 7·6			4·6	3·7, 5·8	4·4	3·4, 5·8	5·4	3·4, 8·5		
Black	1·5	1·1, 2·1	1·5	1·0, 2·3	1·4	0·8, 2·6			1·3	0·8, 1·9	1·2	0·8, 2·0	1·4	0·7, 3·0		
Other & unknown	2·1	1·5, 3·1	2·1	1·3, 3·3	2·2	1·2, 3·9			1·9	1·2, 3·1	1·9	1·0, 3·4	2·1	1·1, 4·2		
Household composition																
No other adults, no children	15·2	13·6, 16·9	15·9	14·1, 18·0	13·0	10·2, 16·4	26·51	<0·0001	16·1	14·2, 18·1	17·1	15·0, 19·5	12·4	9·3, 16·5	22·05	<0·0001
Other adults, no children	52·0	49·7, 54·2	56·5	54·0, 59·1	37·7	33·4, 42·2			52·9	50·3, 55·5	56·9	54·0, 59·7	38·6	33·3, 44·2		
No other adults, with children	5·8	4·8, 7·1	4·0	3·1, 5·2	11·4	8·4, 15·2			5·3	4·1, 6·7	3·4	2·5, 4·7	11·8	8·0, 17·0		
Other adults, with children	27·0	25·0, 29·1	23·5	21·4, 25·8	38·0	33·5, 42·7			25·8	23·6, 28·1	22·6	20·3, 25·1	37·2	31·9, 42·7		
Employment status																
Full time	57·2	55·0, 59·4	57·4	54·8, 59·9	56·6	52·0, 61·2	6·50	<0·0001	58·9	56·3, 61·4	58·5	55·6, 61·3	60·3	54·7, 65·7	5·09	0·004
Part time	18·5	16·8, 20·3	18·5	16·5, 20·5	18·6	15·3, 22·4			18·2	16·3, 20·3	18·4	16·2, 20·7	17·7	13·7, 22·6		
Looking for work	4·7	3·9, 5·7	3·5	2·7, 4·5	8·6	6·4, 11·4			4·1	3·2, 5·2	3·0	2·2, 4·1	8·0	5·5, 11·4		
Not looking for work	19·2	17·5, 21·0	20·5	18·5, 22·6	15·3	12·4, 18·8			18·6	16·7, 20·7	20·0	17·8, 22·4	13·8	10·5, 17·8		
Unknown	0·4	0·2, 0·9	0·3	0·1, 0·9	0·9	0·3, 2·6			0·2	0·04, 0·8	0·2	0·02, 1·2	0·2	0·03, 1·6		
Student status																
No	87·1	85·6, 88·5	90·9	89·4, 92·2	75·4	71·3, 79·1	26·80	<0·0001	87·5	85·8, 89·0	90·8	89·1, 92·3	75·5	70·6, 79·8	20·06	<0·0001
Yes, full time	8·6	7·5, 9·9	5·9	4·8, 7·1	17·1	13·9, 20·8			8·5	7·3, 10·0	5·8	4·7, 7·2	18·2	14·5, 22·6		
Yes, part time	4·1	3·4, 5·1	3·1	2·4, 4·0	7·4	5·4, 10·1			3·8	3·0, 4·9	3·2	2·4, 4·2	6·1	4·0, 9·3		
Unknown	0·1	0·03, 0·6	0·1	0·02, 0·9	0·2	0·02, 1·1			0·2	0·04, 0·8	0·2	0·02, 1·2	0·2	0·03, 1·6		
Making ends meet																
Difficult	22·1	20·2, 24·1	10·4	9·0, 12·1	58·5	53·8, 63·0	160·03	<0·0001	19·6	17·6, 21·7	9·5	8·0, 11·3	55·6	49·9, 61·1	113·85	<0·0001
Neither easy nor difficult	33·4	31·3, 35·6	35·7	33·3, 38·2	26·4	22·5, 30·7			32·6	30·2, 35·0	34·5	31·7, 37·3	25·9	21·3, 31·0		
Easy	44·0	41·8, 46·3	53·3	50·7, 55·9	14·9	11·9, 18·5			47·6	45·0, 50·2	55·7	52·8, 58·5	18·6	14·6, 23·4		
Unknown	0·5	0·3, 0·9	0·6	0·3, 1·1	0·2	0·03, 1·7			0·3	0·1, 0·8	0·4	0·2, 1·0	0			
Education*																
Low	29·0	26·9, 31·1	25·5	23·3, 27·8	39·8	35·3, 44·6	13·10	<0·0001	26·0	23·8, 28·4	23·2	20·8, 25·9	36·1	30·7, 41·8	8·46	<0·0001
Medium	27·2	25·2, 29·3	27·4	25·2, 29·8	26·6	22·6, 30·9			26·9	24·6, 29·2	27·2	24·7, 29·9	25·8	21·3, 30·9		
High	43·4	41·2, 45·6	46·7	44·2, 49·3	33·0	28·8, 37·5			46·8	44·3, 49·4	49·3	36·4, 52·2	38·0	32·7, 43·5		
Unknown	0·5	0·2, 0·9	0·4	0·2, 0·8	0·7	0·2, 2·2			0·3	0·1, 0·6	0·3	0·1, 0·7	0·2	0·02, 1·2		
BMI																
Underweight (<18·5)	N/A		N/A		N/A		N/A		4·6	3·7, 5·8	4·0	3·1, 5·2	6·9	4·6, 10·2	2·39	0·07
Normal (18·5–25)	N/A		N/A		N/A				46·7	44·1, 49·3	47·7	44·8, 50·6	43·1	37·7, 48·7		
Overweight (25·1–30)	N/A		N/A		N/A				31·3	28·9, 33·8	31·7	29·0, 34·5	29·9	24·9, 35·3		
Obese (>30)	N/A		N/A		N/A				17·4	15·5, 19·5	16·6	14·6, 19·0	20·1	15·5, 25·7		

IQR, interquartile range; N/A, not applicable.

* Low = GCSE level or equivalent (UK qualification level 2) and below, medium = A level and NVQ level 4–5 or equivalent (UK qualification level 3–5), high = degree and equivalent (UK qualification level 6) or above.

Incomplete food security status data were mostly due to systematic survey errors, resulting in follow-up questions not being asked of some eligible participants (*n* 599). This was more likely if participants indicated potential FI in one or two, rather than three, of the first three questions. Because of the large number of participants we excluded due to missing food security status, we conducted a sensitivity analysis where we included participants with missing food security status and, in turn, assumed they were food secure or food insecure.

## Results

### Study population

Our main analytical sample included 2551 adults (see Table 1). Overall, 24·3 % of participants were food insecure, including 15·5 % who were classified as having very low food security (see Table 2). A sub-sample was used to examine associations with BMI (*n* 1949). The main sample and BMI sub-sample did not differ significantly in socio-demographic characteristics and, when weighted, were representative of the UK adult population in terms of sex, age and region of residence (see online Supplementary Table S3). However, the samples were somewhat more highly educated and had a lower proportion of overweight than the national population of the UK.

**Table 2:** Weighted proportion of adult food security status (*n* 2551)

Food security classification	Score	Prevalence (%)	Dichotomous food security classification	Prevalence (%)
High food security	0	71·6	Food secure	75·7
Marginal food security	1–2	4·1		
Low food security	3–5	8·8	Food insecure	24·3
Very low food security	6–10	15·5		

### Socio-demographic correlates of food insecurity

#### Descriptive analysis

In the univariable analyses, food insecure adults, compared to food secure adults, were younger (median age 36 *v*. 46 years, *P* < 0·0001) and more likely to be a student (24·5 *v*. 9·0 %, *P* < 0·0001) (see Table 1). Among the food insecure group, there was a higher proportion of Asian and mixed ethnicity participants and lower proportion of white British participants, compared to in the food secure group (*P* < 0·01). Food insecure adults, compared to food secure adults, were also more likely to be living with a child (49·4 *v*. 27·5 %, *P* < 0·0001), particularly in single-parent households. Although food insecure adults were more likely to be looking for work (*P* < 0·0001), compared to food secure adults, the proportion reporting full-time (57 %) and part-time (19 %) employment was similar in both groups. Food insecure adults, compared to food secure adults, were more likely to report difficulty making ends meet (58·5 *v*. 10·4 %, *P* < 0·0001) and have low education (39·8 *v*. 25·5 %, *P* < 0·0001). Food security status did not differ by sex (*P* = 0·35) or BMI (*P* = 0·07).

#### Socio-demographic characteristics of food insecure adults

In the model adjusted for markers of socio-demographic characteristics, including socio-economic variables (model 2), there were higher odds of FI among male participants compared to female participants (OR 1·36; 95 % CI 1·01, 1·83) (see Table 3). Odds of FI decreased with each year of age increase (OR 0·95; 95 % CI 0·94, 0·96). The odds of FI were higher among participants who reported their ethnicity as white other (OR 2·04; 95 % CI 1·04, 3·99) or mixed (OR 2·32; 95 % CI 1·02, 5·27), compared to white British. Participants living with children had higher odds of FI, compared to those living alone, especially if living in a single-parent household (OR 2·10; 95 % CI 1·19, 3·70). Participants who reported not looking for work had lower odds of FI compared to participants who reported being in full-time employment (OR 0·60; 95 % CI 0·42, 0·87). The odds of FI were higher among full-time students compared to non-students (OR 3·23; 95 % CI 2·01, 5·18). Participants reporting difficulty making ends meet had substantially higher odds of FI compared to participants who reported that making ends meet was easy (OR 19·76; 95 % CI 13·78, 28·34). Participants with low education had higher odds of FI compared to those with high education (OR 2·30; 95 % CI 1·66, 3·17).

**Table 3:** Food insecurity by socio-demographic characteristics (*n* 2551)

	Model 1†		Model 2‡	
	OR	95 % CI	OR	95 % CI
Sex				
Female	REF		REF	
Male	1·26*	1·00, 1·60	1·36*	1·01, 1·83
Age (years)	0·95***	0·94, 0·96	0·95***	0·94, 0·96
Ethnicity				
White British	REF		REF	
White other	1·31	0·73, 2·41	2·04*	1·04, 3·99
Mixed	2·33**	1·31, 4·14	2·32*	1·02, 5·27
Asian	1·17	0·68, 2·03	1·69	0·87, 3·29
Black	0·73	0·32, 1·69	0·52	0·14, 1·88
Other & unknown	1·11	0·52, 2·38	1·23	0·52, 2·93
Household composition				
No other adults, no children	REF		REF	
Other adults, no children	0·66*	0·46, 0·93	0·61*	0·41, 0·93
No other adults, with children	3·69***	2·18, 6·25	2·10**	1·19, 3·70
Other adults, with children	1·62**	1·13, 2·32	1·59*	1·05, 2·42
Employment status				
Full time	REF		REF	
Part time	1·15	0·83, 1·60	0·76	0·50, 1·15
Looking for work	2·17**	1·33, 3·52	0·84	0·46, 1·56
Not looking for work	1·07	0·77, 1·47	0·60**	0·42, 0·87
Student status				
No	REF		REF	
Yes, part time	2·06***	1·29, 3·30	1·59	0·80, 3·14
Yes, full time	2·23***	1·56, 3·20	3·23***	2·01, 5·18
Making ends meet				
Easy	REF		REF	
Neither easy nor difficult	2·54***	1·83, 3·53	2·55***	1·83, 3·57
Difficult	20·03***	14·13, 28·38	19·76***	13·78, 28·34
Education				
High	REF		REF	
Medium	1·34*	1·01, 1·79	1·13	0·81, 1·57
Low	2·79***	2·11, 3·69	2·30***	1·66, 3·17

† Model 1: mutually adjusted for sex, age, ethnicity and household composition.

‡ Model 2: mutually adjusted for sex, age, ethnicity, and household composition, employment status, student status, making ends meet and education.

*P ≤ 0·05, **P ≤ 0·01, ***P ≤ 0·001.

### Diet and health

#### Descriptive analysis

Food secure and food insecure adults differed significantly on all diet and health outcomes in the univariable analyses, except for median fruit intake and BMI (see Table 4). In unadjusted analyses, both food secure and food insecure adults had a median fruit intake of once per day, whereas food insecure adults had lower vegetable intake frequency (1·07 *v*. 1·29 times/d, *P* < 0·0001) and higher fruit juice intake frequency (0·39 *v*. 0·29 times/d, *P* = 0·0001). A larger proportion of food insecure adults, compared to food secure adults, reported poor healthiness of diet (46·7 *v*. 33·8 %, *P* < 0·0001), poor general health (42·4 *v*. 29·2 %, *P* < 0·0001), poor mental health (39·7 *v*. 22·2 %, *P* < 0·0001) and high stress (37·3 *v*. 14·3 %, *P* < 0·0001). Approximately half of all the participants were overweight (*P* = 0·62).

**Table 4:** Distribution of outcome measures (with sample weights)

	Overall sample (*n* 2551)		Food secure (*n* 1890)		Food insecure (*n* 661)		Pearson’s *F* statistic	*P*-value
Dietary component								
	Median		Median		Median			
Fruit (times/d)	1·00		1·00		1·00		0·00	1·00
Vegetables (times/d)	1·16		1·29		1·07		37·69	<0·0001
Fruit juice (times/d)	0·29		0·29		0·39		15·62	0·0001
Self-rated diet and health								

	%	95 % CI	%	95 % CI	%	95 % CI		
Healthiness of diet								
Poor	36·9	34·7, 39·1	33·8	31·4, 68·7	46·7	42·0, 51·4	23·84	<0·0001
Good	63·1	60·9, 65·3	66·2	63·7, 68·7	53·3	48·6, 58·0		
General health								
Poor	32·4	30·3, 34·6	29·2	26·9, 31·7	42·4	37·8, 47·1	26·54	<0·0001
Good	67·6	65·4, 69·7	70·8	68·4, 73·1	57·6	52·9, 62·2		
Mental health								
Poor	26·4	24·5, 28·5	22·2	20·1, 24·5	39·7	35·3, 44·3	53·30	<0·0001
Good	73·6	71·5, 75·5	77·8	75·6, 79·9	60·3	55·7, 64·7		
Stress								
High stress	19·9	18·1, 21·8	14·3	12·6, 16·3	37·3	32·8, 42·1	107·19	<0·0001
Low stress	80·1	78·2, 81·9	85·7	83·7, 87·4	62·7	57·9, 67·2		
BMI*								
Not overweight	51·3	48·7, 53·9	51·7	48·7, 54·6	50·0	44·4, 55·7	0·25	0·62
Overweight	48·7	46·1, 51·3	48·3	45·4, 51·3	50·0	44·3, 55·7		

* BMI sub-sample used (*n* 1949: 1495 food secure and 454 food insecure).

#### Frequency of fruit and vegetable intake

In the adjusted models, odds of consuming fruits and vegetables above median frequency were lower in food insecure adults compared to food secure adults (OR 0·59; 95 % CI 0·47, 0·74 and OR 0·68; 95 % CI 0·54, 0·86, respectively), but higher for fruit juice (OR 1·39; 95 % CI 1·10, 1·75) (see Table 5). There were interactions by sex, ethnicity and age, but not household composition (see online Supplementary Table S2). The adjusted odds of fruit intake above median frequency were significantly lower in food insecure adults, compared to food secure adults, across all age groups (ORs ranging from 0·39 to 0·62) except those aged 40–49 and 60–64 years, where the association was not significant. The associations between FI and vegetable and fruit juice intake frequency were not significant in men, but were in women: OR 0·53 (95 % CI 0·39, 0·73) and OR 1·66 (95 % CI 1·21, 2·28), respectively. Age also altered the association between FI and vegetable intake frequency; the association was statistically significant among those aged 31–39 years, OR 0·59 (95 % CI 0·36, 0·98) and 50–59 years, OR 0·35 (95 % CI 0·17, 0·71), but not in other age groups. The association between FI and fruit juice intake frequency was only statistically significant in two ethnic groups (white British and black). These associations were in opposite directions, with higher odds of above median fruit juice intake frequency among food insecure adults than food secure adults who were white British, OR 1·50 (95 % CI 1·16, 1·93), and lower odds for participant who were black, OR 0·11 (95 % CI 0·02, 0·62).

**Table 5:** Achieving intake frequency above the median for fruit, vegetables and fruit juice among food insecure adults†

	Fruit (*n* 2551)		Vegetable (*n* 2551)		Fruit juice (*n* 2551)	
	Adjusted OR	95 % CI	Adjusted OR	95 % CI	Adjusted OR	95 % CI
Overall						
Food secure	REF		REF		REF	
Food insecure	0·59***	0·47, 0·74	0·68**	0·54, 0·86	1·39**	1·10, 1·75
Sex						
Male	N/A		0·86	0·62, 1·20	1·15	0·83, 1·60
Female			0·53***	0·39, 0·73	1·66**	1·21, 2·28
Ethnicity						
White British	N/A		N/A		1·50**	1·16, 1·93
White other					1·55	0·53, 4·51
Mixed					0·74	0·20, 2·75
Asian					1·18	0·44, 3·18
Black					0·11*	0·02, 0·62
Other & unknown					4·20	0·60, 29·16
Age groups						
18–24	0·55*	0·31, 0·96	0·61	0·34, 1·11	N/A	
25–30	0·62**	0·45, 0·85	1·02	0·74, 1·41		
31–39	0·51**	0·31, 0·86	0·59*	0·36, 0·98		
40–49	0·70	0·42, 1·17	0·83	0·50, 1·38		
50–59	0·39**	0·21, 0·75	0·35**	0·17, 0·71		
60–64	0·62	0·19, 2·00	0·56	0·17, 1·83		
Household composition						
No other adults, no children	N/A		N/A		N/A	
Other adults, no children						
No other adults, with children						
Other adults, with children						

N/A, not applicable because no significant interaction was detected.

† Logistic regression models mutually adjusted for sex, age, ethnicity and household composition.

* *P* ≤ 0·05, **P ≤ 0·01, ***P ≤ 0·001.

#### Healthiness of diet and health

Food insecure adults had higher adjusted odds of reporting unhealthy diets compared to food secure adults (OR 1·65; 95 % CI 1·31, 2·09) (see Table 6). Food insecure participants also had higher odds of reporting poor general health (OR 1·90; 95 % CI 1·50, 2·41). This association was statistically significant in all age groups, except for 18–24 and 50–59 years. Food insecure participants also had higher adjusted odds of reporting poorer mental health (OR 2·10; 95 % CI 1·64, 2·69) and high stress (OR 3·15; 95 % CI 2·42, 4·11). The strength of these associations increased with age. The association with mental health also differed by household composition, as it was not statistically significant for participants living alone, but was significant for other household composition categories. Additionally, in the BMI sub-sample, food insecure adults had higher odds of overweight compared to food secure adults (OR 1·32; 95 % CI 1·00, 1·75). This association appeared to be stronger in women than in men, but once stratified, the CI crossed one and became statistically non-significant. The association with BMI only reached statistical significance in the 40–49 years age group.

**Table 6:** Self-reported healthiness of diet and health outcomes among food in secure adults†

	Poor healthiness of diet (*n* 2551)		Poor general health (*n* 2551)		Poor mental health (*n* 2551)		High stress (*n* 2551)		Overweight (*n* 1949)	
	Adjusted OR	95 % CI	Adjusted OR	95 % CI	Adjusted OR	95 % CI	Adjusted OR	95 % CI	Adjusted OR	95 % CI
Overall										
Food secure	REF		REF		REF		REF		REF	
Food insecure	1·65***	1·31, 2·09	1·90***	1·50, 2·41	2·10***	1·64, 2·69	3·15***	2·42, 4·11	1·32*	1·00, 1·75
Sex										
Male	N/A		N/A		N/A		N/A		1·30	0·88, 1·91
Female									1·36	0·91, 2·04
Ethnicity										
White British	N/A		N/A		N/A		N/A		N/A	
White other										
Mixed										
Asian										
Black										
Other & unknown										
Age groups (years)										
18–24	N/A		1·21	0·64, 2·28	1·45	0·79, 2·65	3·15**	1·57, 6·33	1·12	0·49, 2·56
25–30			2·16***	1·54, 3·02	2·00***	1·41, 2·82	2·44***	1·68, 3·52	1·42	0·97, 2·08
31–39			2·04**	1·18, 3·52	2·34**	1·32, 4·13	3·43***	1·87, 6·29	1·34	0·70, 2·58
40–49			2·61***	1·53, 4·45	2·33**	1·35, 4·00	3·14***	1·79, 5·51	2·16*	1·14, 4·06
50–59			1·63	0·89, 2·99	2·39**	1·24, 4·58	3·80***	1·97, 7·36	0·76	0·35, 1·68
60–64			4·56*	1·17, 17·74	17·10***	3·72, 78·56	8·43***	2·50, 28·47	1·04	0·32, 3·38
Household composition										
No other adults, no children	N/A		N/A		1·79	0·94, 3·39	N/A		N/A	
Other adults, no children					2·15***	1·49, 3·08				
No other adults, with children					2·57*	1·05, 6·29				
Other adults, with children					2·22***	1·43, 3·45				

N/A, not applicable because no significant interaction was detected.

† Logistic regression models adjusted for sex, age, ethnicity and household composition.

*P ≤ 0·05, **P ≤ 0·01, ***P ≤ 0·001.

### Sensitivity analysis

Adjusting for additional markers of socio-economic position altered some of our findings. The associations between socio-demographic characteristics and FI were similar in models 1 and 2 (see Table 3). However, adjusting for employment status, student status, ability to make ends meet and education did remove the association between looking for work and FI. The associations between food security status and fruit, vegetable and fruit juice intake frequencies did not change (see online Supplementary Table S5). However, the associations with self-reported healthiness of diet, general health, mental health and overweight were no longer statistically significant (see online Supplementary Table S6). The association with self-reported stress, however, remained strong (OR 2·16; 95 % CI 1·59, 2·95).

When we assumed that all participants with missing adult food security status were food secure (or food insecure), the weighted prevalence of FI was 20·6 % (or 43·6 %). The true value is likely to be somewhere in between.

## Discussion

We found that the prevalence of FI was 24·3 % among a national sample of UK adults, which is higher than previous estimates in the UK. Participants reporting that making ends meet was difficult compared to easy had almost twenty times the odds of FI, when adjusted for other socio-demographic characteristics. The adjusted odds of FI were higher in males compared to females, those who reported their ethnicity as white other or mixed compared to white British, full-time students compared to non-students, and participants with low compared to high education. Participants with children, especially in single-parent households, had higher adjusted odds of FI compared to those living alone. Younger adults also had higher adjusted odds of FI compared to older adults. We found food insecure adults to have lower adjusted odds of consuming above the median frequency for fruits and vegetables and higher adjusted odds of consuming fruit juice, compared to food secure adults. We also found that food insecure adults had higher odds of reporting poor healthiness of diet, general health, and mental health, as well as high stress and overweight, compared to food secure adults. Together, these findings highlight the high prevalence of FI in the UK, especially among some socio-economically disadvantaged groups, and add to the evidence for associations between FI and poorer diet and health. However, the cross-sectional nature of this study limits our interpretation of these associations.

### Comparison of results to other studies

Difficulty in making ends meet, younger age, having children and low education were found to be associated with FI in the UK in our study, consistent with previous work^(3)^. Similar to our findings, other studies have also found that food insecure adults consumed fewer fruits and vegetables and had less healthy diets in general, compared to food secure adults^(14)^. We also observed poorer self-reported physical and mental health, and high self-reported stress among food insecure adults, which is consistent with other studies^(15,33–35)^. Power and colleagues found that FI was associated with poor health in UK mothers, but this was not significant when adjustment for perceived financial situation was made^(21)^. In our study, associations between FI and health were also extinguished once socio-economic factors were adjusted for, with the exception of the association with high self-reported stress. The attenuation of these associations suggests that part of the association between FI and these outcomes was due to covariance of FI with socio-economic factors. Socio-economic characteristics were associated with FI in our adjusted models, with those reporting that making ends meet was difficult having almost twenty times higher adjusted odds of FI compared to those reporting that making ends meet was easy.

The Food and You Survey (wave 4, 2016) reported prevalence of adult FI (measured by AFSSM) in the UK as 8 %^(2)^, which was lower than we observed. The difference could be due to differences in socio-demographic characteristics between the two samples. Unlike the current work, the Food and You Survey included participants aged 16–18 years and over 65 years. In the Food and You Survey, prevalence of FI was lowest in over 65 year olds (1–2 %), which represented 22 % of the sample. Participants may also be more willing to disclose FI in anonymised online surveys (IFPS) than in face-to-face interviews (Food and You Survey).

### Interpretation of findings and implications for policy

Reported difficulty in making ends meet had the strongest association with FI in our adjusted models. With rising prices of relatively inflexible necessities, such as the 45 % rise in fuel costs in the UK over the last decade^(6)^, pressure has been put on household budgets. This may be at the expense of diet quality. FI was associated with poorer self-rated healthiness of diet, suggesting that food insecure adults were aware of their poor diet. We observed higher fruit juice intake among food insecure adults compared to food secure adults, an association also reported in the United States^(36)^. Fruit juice may be preferred by food insecure adults under economic constraints, as fruit juice is cheaper than the equivalent whole fruit^(37)^. Although fruit juice can count as one portion of fruit per day according to the UK’s 5-a-day recommendation, it is a major source of free sugars. The World Health Organisation (WHO) recommends limiting free sugar intake to no more than 10 % of total energy intake, with further benefits from reducing to <5 %^(38)^. Thus, the additional fruit juice consumed by food insecure adults could have a negative cumulative health effect.

FI was associated with poorer health outcomes, especially high self-reported stress and poor mental health, pointing to a strong correlation between FI and mental well-being. These findings are in line with previous research from elsewhere^(20,35)^ and supported by research that found FI and food bank use to be stigmatising, isolating and shameful for those experiencing FI^(39,40)^. Although FI was less prevalent in older adults compared to younger adults, the association with poor health outcomes appeared stronger in the older age groups, especially for poor mental health and high stress. The persisting association between high self-reported stress and FI in this study even after adjustments for socio-economic variables suggests that this association is specific to FI, over and above socio-economic deprivation. Further studies are needed to determine the causality of these associations and, if so, mechanisms driving them. Nonetheless, regardless of the direction of association, we must acknowledge the stressful lives of those experiencing FI. Many food insecure individuals report experiencing adverse life events and financial strain^(26)^. Food insecure adults had higher odds of overweight compared to food secure adults. Reliance on cheap energy-dense foods in favour of nutrient-dense foods such as fruit and vegetables is likely to be a common coping strategy when facing FI^(12)^, leading to compromised diet quality but not necessarily reduced energetic intake.

The UK has a high prevalence of individual-level FI relative to its poverty rate, compared to other European countries, which may be related to the UK’s wide income inequality^(41)^. The suboptimal diet of the UK population as a whole, and especially in lower socio-economic groups^(42)^, points to a need for structural changes to the food, economic and welfare systems. Addressing the high and rising cost of food, especially healthy foods^(10)^, could be one important approach. We observed that FI was more likely in participants who reported difficulty making ends meet. Unemployment and delayed social benefit payments are frequently cited reasons for using food banks^(26)^. However, FI is not just a problem among unemployed individuals, as 76 % of the food insecure adults in our sample reported being employed. People working full-time on the National Living Wage do not necessarily achieve the Minimum Income Standard – the income needed to reach a minimum socially acceptable standard of living^(6)^. This points to the UK welfare system and wage-related policies being insufficient to protect all members of society from FI, and its potential impacts on physical and mental health.

Whilst structural changes may be the most effective way to address FI, these are politically contentious and have long policy timelines. In the meantime, interventions that address the symptoms of FI, including hunger and poor diet quality, could help to alleviate the immediate impacts. The Trussell Trust provided 1·6 million emergency food parcels in 2018/2019^(4)^. The government’s Healthy Start programme, which provides expectant mothers and mothers of young children on low incomes with vouchers to purchase milk, fruits and vegetables^(43)^, could also reduce hunger and improve diet quality. However, the scheme has benefited fewer individuals than intended, due to low uptake^(43)^. Reported barriers to uptake include stigma surrounding voucher use, complexity related to the application process, receipt of vouchers and use, and lack of awareness^(43)^. Over time, the real value of Healthy Start vouchers has also diminished, from £2·80 in 1992 (equivalent to £5·69 in 2018) when the scheme started, to £3·10 today^(44)^. Increasing the uptake and value of this scheme may be particularly valuable as FI is more prevalent in adults living with children, compared to adults living alone. FI was also higher among younger adults and students, who may benefit from targeted interventions.

### Strengths and limitations

This national study sample, when weighted, was representative of the UK adult population in terms of sex, age and region of residence (see online Supplementary Table S3), providing a unique opportunity to estimate FI prevalence and explore correlates of FI in a general UK adult population. To our knowledge, this is the first study in the UK to explore associations between adult food security and diet and health in a general population sample. However, excluding participants with missing adult food security status may have introduced selection bias. Nonetheless, our sensitivity analysis estimated FI prevalence at between 20·6 and 43·6 % in our sample. Even the conservative estimate of 20·6 % indicates a high prevalence that cannot be ignored.

The AFSSM is a validated measure of adult food security^(28)^; however, it focuses on food adequacy. The scale does not capture other elements of food security: preferences, safety, or nutrition, with only one question related to ‘balanced meals’. The Behavioural Risk Factor Surveillance System fruit and vegetable module has moderate validity and reliability when compared to reference dietary assessment methods^(29)^. Unfortunately, more detailed dietary assessment was not included in the IFPS. Future work could explore associations between FI and more holistic markers of diet quality. Self-rated health provides a validated proxy of actual health^(45)^, and moderate associations have been found between self-rated mental health and validated mental health scales^(46)^. However, as with all self-reported data, these data may be subject to social desirability bias.

### Future research

Routine measurement of food security, rather than just food bank usage data, in the UK population would help confirm the relationships we have reported and track prevalence, determinants and outcomes of FI over time. Since our analysis was conducted, the UK government has announced that FI will be routinely measured from April 2019 in the annual Family Resources Survey^(47)^. This will also allow the impact of planned and unplanned interventions that may influence food security to be evaluated.

## Conclusions

FI was prevalent among UK adults and correlated with various socio-demographic characteristics. Reported difficulty in making ends meet had the strongest association with FI. FI was also associated with poorer diet and health, as measured by a number of markers. FI is unlikely to be a healthful experience and may be both influenced by, and lead to, poor physical and mental health.

## Supplementary material

For supplementary material accompanying this paper visit https://doi.org/10.1017/S1368980020000087. click here to view supplementary material
