# Supplementary material for: Socio-demographic characteristics, diet and health among food insecure UK adults: cross-sectional analysis of the International Food Policy Study
Source: Public Health Nutr. 2020 Apr 27;23(14):2602–14. doi: 10.1017/S1368980020000087 (PMC7116035; doi:10.1017/S1368980020000087)
Supplement: Supplementary file 1 [file S1368980020000087sup001.docx]

**SUPPLEMENTARY MATERIAL**

**Supplementary Table S1.** Questions used to assess food security status (adapted from USDA’s Adult Food Security Survey Module)

|  | **Question** | **Responses** | **Score** |
| --- | --- | --- | --- |
| HH1 | Which of these statements best describes the food eaten in your household in the last 12 months: | You and other household members always had enough of the kinds of foods you wanted to eat.  You and other household members had enough to eat, but not always the kinds of food you wanted.  Sometimes you and other household members did not have enough to eat.  Often you and other household members didn't have enough to eat.  Don’t know  Refuse to answer | N/A |
| HH2 | Now you will see several statements that may be used to describe the food situation for a household. Please indicate if the statement was often true, sometimes true, or never true for you and other household members IN THE PAST 12 MONTHS.  You and other household members worried that food would run out before you got money to buy more. | Often true  Sometimes true  Never true  Don’t know  Refuse to answer | 1  1  0  0  N/A |
| HH3 | The food that you and other household members bought just didn't last, and there wasn't any money to get more. | Often true  Sometimes true  Never true  Don’t know  Refuse to answer | 1  1  0  0  N/A |
| HH4 | You and other household members couldn't afford to eat balanced meals. | Often true  Sometimes true  Never true  Don’t know  Refuse to answer | 1  1  0  0  N/A |
| If affirmative response (i.e., "often true" or "sometimes true") to one or more of Questions HH2-HH4, OR, response [3] or [4] to question HH1 (if administered), then continue to Adult Stage 2 | | | |
| AD1 | In the last 12 months, since last (name of current month), did you or other adults in your household ever reduce the size of your meals or skip meals because there wasn't enough money for food? | Yes  No  Don’t know  Refuse to answer | 1  0  0  N/A |
| AD1a | How often did this happen—almost every month, some months but not every month, or in only 1 or 2 months? | Almost every month  Some months but not every month  Only 1 or 2 months  Don’t know  Refuse to answer | 1  1  0  0  N/A |
| AD2 | In the last 12 months, did you ever eat less than you felt you should because there wasn't enough money for food? | Yes  No  Don’t know  Refuse to answer | 1  0  0  N/A |
| AD3 | In the last 12 months, were you every hungry but didn't eat because there wasn't enough money for food? | Yes  No  Don’t know  Refuse to answer | 1  0  0  N/A |
| AD4 | In the last 12 months, did you lose weight because there wasn't enough money for food? | Yes  No  Don’t know  Refuse to answer | 1  0  0  N/A |
| If affirmative response to one or more of questions AD1 through AD4, then continue to Adult Stage 3; otherwise skip to End of Food Security Module. | | | |
| AD5 | In the last 12 months, did you or other adults in your household ever not eat for a whole day because there wasn't enough money for food? | Yes  No  Don’t know  Refuse to answer | 1  0  0  N/A |
| AD5a | How often did this happen—almost every month, some months but not every month, or in only 1 or 2 months? | Almost every month  Some months but not every month  Only 1 or 2 months  Don’t know  Refuse to answer | 1  1  0  0  N/A |

**Supplementary Table S2:** F statistic (*P*-value) for interactions between sociodemographic characteristics and food security status on association with diet and health

| **Interaction** | **Fruit** | **Vegetables** | **Juice** | **Diet** | **General health** | **Mental health** | **Stress** | **BMI** |
| --- | --- | --- | --- | --- | --- | --- | --- | --- |
| Adult Food Security#Sex | 1.40  (0.24) | 9.50  (<0.0001)*** | 7.08  (0.0001)*** | 1.74  (0.16) | 2.45  (0.06) | 2.86  (0.04) | 0.90  (0.44) | 7.75  (<0.0001)*** |
| Adult Food Security#Age | 8.34  (<0.0001)*** | 2.58  (0.05)* | 1.57  (0.19) | 1.96  (0.12) | 2.63  (0.05)* | 11.84  (<0.0001)*** | 7.05  (0.0001)*** | 32.03  (<0.0001)*** |
| Adult Food Security#Ethnicity | 0.96  (0.48) | 1.56  (0.11) | 2.16  (0.01)** | 1.62  (0.09) | 1.17  (0.30) | 1.06  (0.39) | 0.67  (0.77) | 1.02  (0.43) |
| Adult Food Security#Household composition | 0.85  (0.55) | 1.15  (0.33) | 1.45  (0.18) | 0.55  (0.80) | 1.29  (0.25) | 3.33  (0.002)** | 1.44  (0.18) | 0.88  (0.53) |
| * P<0·05, **P<0·01, ***P<0·001 | | | | | | | | |

**Supplementary Table S3:** Characteristics of the full analytical sample, BMI sub-sample, and the UK population

|  | **Overall sample** | **BMI sub-sample** | **Overall sample vs BMI sub-sample**  Pearson’s F Statistic  (*P*-Value) | **UK population**  [source] |
| --- | --- | --- | --- | --- |
| **Food Security**, % (95% CI where available) | | | | |
| Food Secure | 75·7 (73·7, 77·6) | 78·2 (76·0, 80·3) | 2·74  (0·10) | 92 [Food & You, 2017] |
| Food Insecure | 24·3 (22·4, 26·3) | 21·8 (19·7, 24·1) |  | 8 [Food & You, 2017] |
| **Sex**, % (95% CI where available) | | | | |
| Male | 48·9 (46·7, 51·2) | 51·3 (48·7, 53·9) | 1·81  (0·18) | 49·3 [2016 mid-year estimates] |
| Female | 51·1 (48·8, 53·3) | 48·7 (46·1, 51·3) |  | 50·7 [2016 mid-year estimates] |
| **Age**, median (IQR) | 44 (32, 54) | 45 (32, 55) | 1·80  (0·18) | 41 (22, 53) [2016 mid-year estimates] |
| **Ethnicity**, % (95% CI where available) |  |  |  |  |
| White British | 85·2 (83·5, 86·7) | 84·2 (82·2, 86·0) | 0·34  (0·88) | 80·5 [Census 2011] |
| White other | 4·6 (3·8, 5·7) | 5·2 (4·2, 6·4) |  | 5·5 [Census 2011] |
| Mixed | 2·5 (1·9, 3·3) | 2·8 (2·1 , 3·8) |  | 2·2 [Census 2011] |
| Asian | 4·1 (3·3, 5·1) | 4·6 (3·7, 5·8) |  | 7·5 [Census 2011] |
| Black | 1·5 (1·1, 2·1) | 1·3 (0·8, 1·9) |  | 3·3 [Census 201] |
| Other & unknown | 2·1 (1·5, 3·1) | 1·9 (1·2, 3·1) |  | 1·0 [Census 2011] |
| **Household composition**, % (95% CI where available) | | | | |
| No other adults, no children | 15·2 (13·6, 16·9) | 16·1 (14·2, 18·1) | 0·45  (0·72) | 28.3 [Families and Households 2017] |
| Other adults, no children | 52·0 (49·7, 54·2) | 52·9 (50·3, 5·5) |  | 31.8 [Families and Households 2017] |
| No other adults, with children | 5·8 (4·8, 7·1) | 5·3 (4·1, 6·7) |  | 6.2 [Families and Households 2017] |
| Other adults, with children | 27·0 (25·0, 29·1) | 25·8 (23·6, 28·1) |  | 30.1 [Families and Households 2017] |
| **Employment^a^**, % (95% CI where available) | | | | |
| Full time | 57·2 (55·0, 59·4) | 58·9 (56·3, 61·4) | 0·63  (0·64) | 74·5^b^ [UK Labour Market Jan 2017] |
| Part time | 18·5 (16·8, 20·3) | 18·2 (16·3, 20·3) |  |  |
| Looking for work | 4·7 (3·9, 5·7) | 4·1 (3·2, 5·2) |  | 4·8 [UK Labour Market Jan 2017] |
| Not looking for work | 19·2 (17·5, 21·0) | 18·6 (16·7, 20·7) |  | 21·7^c^ [UK Labour Market Jan 2017] |
| Unknown | 0·4 (0·2, 0·9) | 0·2 (0·04, 0·8) |  | N/A |
| **Student status**, % (95% CI where available) | | | | |
| No | 87·1 (85·6, 88·5) | 87·5 (85·8, 89·0) | 0·10  (0·95) | Data unavailable |
| Yes, full time | 8·6 (7·5, 9·9) | 8·5 (7·3, 10·0) |  | 7 [Census 2011] |
| Yes, part time | 4·1 (3·4, 5·1) | 3·8 (3·0, 4·9) |  | 2·3 [Universities UK 2015^d^] |
| Unknown | 0·1 (0·03, 0·6) | 0·2 (0·04, 0·8) |  | N/A |
| **Making ends meet**, % (95% CI where available) | | | | |
| Difficult | 22·1 (20·2, 24·1) | 19·6 (17·6, 21·7) | 1·93  (0·12) | 12 ^e^ [YouGov DebtTrack 2013^f^] |
| Neither easy nor difficult | 33·4 (31·3, 35·6) | 32·6 (30·2, 35·0) |  | 40^g^ [YouGov DebtTrack 2013] |
| Easy | 44·0 (41·8, 46·3) | 47·6 (45·0, 50·2) |  | 43^h^ [YouGov DebtTrack 2013] |
| Unknown | 0·5 (0·3, 0·9) | 0·3 (0·1, 0·8) |  | 5 [YouGov DebtTrack 2013] |
| **Education**, % (95% CI where available) | | | | |
| Low | 29·0 (26·9, 31·1) | 26·0 (23·8, 28·4) | 1·95  (0·12) | 49·7 [Census 2011] |
| Medium | 27·2 (25·2, 29·3) | 26·9 (24·6, 29·2) |  | 12·2 [Census 2011] |
| High | 43·4 (41·2, 45·6) | 46·8 (44·3, 49·4) |  | 28·6 [Census 2011] |
| Unknown | 0·5 (0·2, 0·9) | 0·3 (0·1, 0·6) |  | N/A |
| **Body mass index**, % (95% CI where available) | | | | |
| Underweight | N/A | 4·6 (3·7, 5·8) | N/A | 2 [Health Survey for England 2016] |
| Normal | N/A | 46·7 (44·1, 49·3) |  | 37 [Health Survey for England 2016] |
| Overweight | N/A | 31·3 (28·9, 33·8) |  | 35 [Health Survey for England 2016] |
| Obese | N/A | 17·4 (15·5, 19·5) |  | 26 [Health Survey for England 2016] |
| Unknown | N/A | N/A |  | N/A |
| ^a^ ages 16-64 years included in the UK Labour Market January 2017  ^b^ unemployed  ^c^ inactive  ^d^ estimated from Patterns and Trends in UK Higher Education 2015, Universities UK: <https://www.universitiesuk.ac.uk/policy-and-analysis/reports/Documents/2015/patterns-and-trends-2015.pdf>  ^e^ Keeping up with bills and credit commitments is a heavy burden  ^f^ subject indicator of financial difficulties for Credit, Debt and Financial Difficulty in Britain 2012, Department for Business Innovation and Skills: <https://assets.publishing.service.gov.uk/government/uploads/system/uploads/attachment_data/file/208075/bis-13-p187-a-report-using-data-from-the-yougov-debttrack-survey.pdf>  ^g^ Keeping up with bills and credit commitments is somewhat of a burden  ^h^ Keeping up with bills and credit commitments is not a burden at all | | | | |

**Supplementary Table S4:** Odds ratio (95% CI) from adjusted logistic regression models for association between sociodemographic characteristics and diet and health outcomes

| **Sociodemographic characteristic** | | **Fruit** | **Vegetable** | **Fruit juice** | **Healthiness of diet** | **General health** | **Mental health** | **Stress** | **BMI** |
| --- | --- | --- | --- | --- | --- | --- | --- | --- | --- |
| **Sex** | Male | REF | REF | REF | REF | REF | REF | REF | REF |
|  | Female | 1·17  (0·97, 1·42) | 1·53  (1·27, 1·84)*** | 0·66  (0·54, 0·79) | 0·99  (0·81, 1·20) | 0·99  (0·81, 1·22) | 1·39  (1·12, 1·74)** | 1·17  (0·91, 1·50) | 0·59  (0·48, 0·74)*** |
| **Age** | | 1·02  (1·01, 1·02)*** | 1·00  (1·00, 1·01) | 0·99  (0·99, 1·00) | 0·99  (0·99, 1·00) | 1·00  (1·00, 1·01) | 0·98  (0·97, 0·99)*** | 0·99  (0·98, 1·00)* | 1·04  (1·03, 1·05)*** |
| **Ethnicity** | White British | REF | REF | REF | REF | REF | REF | REF | REF |
|  | White other | 1·28  (0·80, 2·05) | 1·16  (0·73, 1·85) | 1·31  (0·85, 2·01) | 0·91  (0·56, 1·46) | 0·54  (0·31, 0·96)* | 0·67  (0·37, 1·20) | 1·19  (0·68, 2·10) | 1·15  (0·72, 1·84) |
|  | Mixed | 1·14  (0·65, 2·00) | 0·91  (0·51, 1·63) | 1·27  (0·70, 2·30) | 0·65  (0·34, 1·26) | 0·58  (0·29, 1·16) | 0·99  (0·50, 1·93) | 0·95  (0·43, 2·08) | 1·71  (0·88, 3·30) |
|  | Asian | 1·10  (0·69, 1·75) | 1·12  (0·69, 1·80) | 1·04  (0·66, 1·64) | 1·10  (0·69, 1·75) | 0·99  (0·61, 1·60) | 0·64  (0·38, 1·05) | 0·69  (0·36, 1·31) | 1·10  (0·65, 1·86) |
|  | Black | 0·97  (0·49, 1·94) | 0·98  (0·49, 1·99) | 1·11  (0·53, 2·34) | 0·37  (0·15, 0·92)* | 0·65  (0·28, 1·55) | 0·39  (0·14, 1·09) | 0·37  (0·14, 0·99)* | 2·02  (0·77, 5·29) |
|  | Other & unknown | 0·95  (0·42, 2·14) | 0·90  (0·44, 1·88) | 1·10  (0·51, 2·38) | 1·69  (0·75, 3·80) | 1·21  (0·49, 3·00) | 0·90  (0·28, 2·93) | 0·71  (0·30, 1·67) | 0·87  (0·29, 2·58) |
| **Household composition** | No other adults, no children | REF | REF | REF | REF | REF | REF | REF | REF |
|  | Other adults, no children | 1·21  (0·91, 1·61) | 0·92  (0·71, 1·21) | 1·16  (0·88, 1·53) | 0·79  (0·59, 1·04) | 0·70  (0·53, 0·93)* | 0·57  (0·42, 0·78)*** | 0·75  (0·52, 1·08) | 1·12  (0·81, 1·54) |
|  | No other adults, with children | 1·55  (0·93, 2·57) | 0·84  (0·53, 1·33) | 1·11  (0·67, 1·82) | 0·97  (0·59, 1·57) | 0·72  (0·44, 1·20) | 0·84  (0·50, 1·39) | 1·22  (0·67, 2·20) | 1·69  (0·93, 3·06) |
|  | Other adults, with children | 1·27  (0·93, 1·75) | 0·79  (0·59, 1·07) | 1·52  (1·12, 2·08) | 0·82  (0·60, 1·13) | 0·69  (0·50, 0·95) * | 0·46  (0·32, 0·65)*** | 0·85  (0·57, 1·27) | 1·23  (0·86, 1·76) |
| N/A, not applicable.  * P<0·05, **P<0·01, ***P<0·001 | | | | | | | | | |

**Supplementary Table S5:** Odds ratio (95% CI) from sensitivity analysis for adjusted logistic regression models looking at association between adult food security and frequency of fruit, fruit juice, and vegetable intake with additional adjustment for socioeconomic variables

|  | | **Fruit**  (*n*=2551) | **Vegetable**  (*n*=2551) | **Fruit Juice**  (*n*=2551) |
| --- | --- | --- | --- | --- |
| **Adult food insecurity** | Food secure | REF | REF | REF |
|  | Food insecure | 0·63 (0·48, 0·82)** | 0·72 (0·54, 0·95)* | 1·45 (1·11, 1·89)** |
| **Sex** | Male | REF | REF | REF |
|  | Female | 1·14 (0·93, 1·40) | 1·50 (1·23, 1·82)*** | 0·70 (0·57, 0·86)** |
| **Age** | | 1·02 (1·01, 1·03)*** | 1·01 (1·00, 1·01) | 1·00 (0·99, 1·01) |
| **Ethnicity** | White British | REF | REF | REF |
|  | White other | 1·23 (0·75, 2·00) | 1·05 (0·65, 1·68) | 1·25 (0·81, 1·93) |
|  | Mixed | 1·05 (0·59, 1·85) | 0·83 (0·46, 1·05) | 1·17 (0·64, 2·13) |
|  | Asian | 1·02 (0·63, 1·64) | 0·99 (0·62, 1·59) | 1·02 (0·65, 1·60) |
|  | Black | 0·96 (0·49, 1·89) | 0·91 (0·41, 2·02) | 1·05 (0·50, 2·24) |
|  | Other & unknown | 1·02 (0·41, 2·57) | 0·92 (0·43, 1·99) | 0·87 (0·35, 2·14) |
| **Household composition** | No other adults, no children | REF | REF | REF |
|  | Other adults, no children | 1·21 (0·91, 1·62) | 0·93 (0·71, 1·23) | 1·17 (0·88, 1·55) |
|  | No other adults, with children | 1·68 (1·00, 2·81) | 0·94 (0·59, 1·48) | 1·19 (0·72, 1·97) |
|  | Other adults, with children | 1·31 (0·95, 1·80) | 0·81 (0·59, 1·10) | 1·58 (1·16, 2·17)** |
| **Employment status** | Full time | REF | REF | REF |
|  | Part time | 1·22 (0·93, 1·59) | 1·11 (0·86, 1·44) | 0·82 (0·63, 1·07) |
|  | Looking for work | 1·02 (0·65, 1·60) | 1·21 (0·78, 1·86) | 0·77 (0·49, 1·21) |
|  | Not looking for work | 1·06 (0·81, 1·38) | 1·14 (0·88, 1·48) | 0·81 (0·63, 1·05) |
| **Student status** | No | REF | REF | REF |
|  | Yes, full time | 1·11 (0·77, 1·59) | 1·23 (0·84, 1·80) | 1·48 (1·04, 2·10)* |
|  | Yes, part time | 1·04 (0·65, 1·64) | 1·42 (0·92, 2·20) | 1·62 (1·03 , 2·56)* |
| **Making ends meet** | Difficult | REF | REF | REF |
|  | Neither easy nor difficult | 0·84 (0·63, 1·12) | 0·88 (0·66, 1·17) | 1·03 (0·78, 1·37) |
|  | Easy | 1·01 (0·75, 1·35) | 1·00 (0·74, 1·33) | 1·23 (0·92, 1·65) |
| **Education** | Low | REF | REF | REF |
|  | Medium | 1·10 (0·85, 1·43) | 1·32 (1·02, 1·71)* | 0·81 (0·62, 1·04) |
|  | High | 1·58 (1·24, 2·01)*** | 1·88 (1·48, 2·38)*** | 0·97 (0·76, 1·23) |
| Logistic regression models mutually adjusted for sex, age, ethnicity, household composition, employment status, student status, ability, make ends meet, and educational level.  * P<0·05, **P<0·01, ***P<0·001 | | | | |

**Supplementary Table S6:** Odds ratio (95%CI) from sensitivity analysis for adjusted logistic regression models looking at association between adult food security and self-reported healthiness of diet and health with additional adjustment for socioeconomic variables

|  | | **Poor healthiness of diet**  (*n*=2551) | **Poor general health**  (*n*=2551) | **Poor mental health**  (*n*=2551) | **High stress**  (*n*=2551) | **Overweight**  (*n*=1949) |
| --- | --- | --- | --- | --- | --- | --- |
| **Adult food insecurity** | Food secure | REF | REF | REF | REF | REF |
|  | Food insecure | 1·17 (0·89, 1·53) | 1·15 (0·88, 1·51) | 1·09 (0·83, 1·45) | 2·16 (1·59, 2·95)*** | 1·28 (0·93, 1·77) |
| **Sex** | Male | REF | REF | REF | REF | REF |
|  | Female | 0·95 (0·77, 1·17) | 0·86 (0·69, 1·07) | 1·23 (0·98, 1·55) | 1·30 (0·99, 1·69) | 0·56 (0·45, 0·71)*** |
| **Age** | | 0·99 (0·98, 1·00)** | 1·00 (0·99, 1·00) | 0·97 (0·97, 0·98)*** | 0·99 (0·98, 1·00)* | 1·03 (1·02, 1·04)*** |
| **Ethnicity** | White British | REF | REF | REF | REF | REF |
|  | White other | 1·01 (0·61, 1·67) | 0·63 (0·36, 1·10) | 0·80 (0·46, 1·42) | 1·20 (0·68, 2,12) | 1·15 (0·71, 1·84) |
|  | Mixed | 0·82 (0·44, 1·53) | 0·75 (0·39, 1·45) | 1·22 (0·64, 2·35) | 1·07 (0·50, 2·28) | 1·93 (0·99, 3·75) |
|  | Asian | 1·29 (0·81, 2·07) | 1·20 (0·73, 1·96) | 0·74 (0·44, 1·26) | 0·70 (0·37, 1·32) | 1·12 (0·65, 1·92) |
|  | Black | 0·39 (0·15, 1·01) | 0·71 (0·29, 1·77) | 0·37 (0·12, 1·17) | 0·34 (0·12, 0·93)* | 2·17 (0·77, 6·08) |
|  | Other & unknown | 2·30 (0·97, 5·44) | 1·58 (0·60, 4·20) | 1·36 (0·39, 4·78) | 0·91 (0·37, 2·26) | 0·65 (0·21, 2·02) |
| **Household composition** | No other adults, no children | REF | REF | REF | REF | REF |
|  | Other adults, no children | 0·83 (0·62, 1·11) | 0·74 (0·54, 1·00) | 0·57 (0·41, 0·78)*** | 0·79 (0·54, 1·16) | 1·18 (0·85, 1·63) |
|  | No other adults, with children | 0·83 (0·50, 1·39) | 0·60 (0·35, 1·03) | 0·67 (0·39, 1·17) | 1·20 (0·68, 2·13) | 1·59 (0·87, 2·91) |
|  | Other adults, with children | 0·83 (0·60, 1·16) | 0·73 (0·52, 1·03) | 0·47 (0·32, 0·67)*** | 0·83 (0·54, 1·26) | 1·25 (0·87, 1·79) |
| **Employment status** | Full time | REF | REF | REF | REF | REF |
|  | Part time | 0·94 (0·71, 1·24) | 1·04 (0·78, 1·39) | 1·08 (0·80, 1·46) | 0·48 (0·34, 0·68)*** | 1·01 (0·74, 1·38) |
|  | Looking for work | 1·38 (0·88, 2·18) | 1·74 (1·10, 2·78)* | 1·81 (1·13, 2·91)* | 0·71 (0·42, 1·19) | 1·10 (0·62, 1·93) |
|  | Not looking for work | 1·26 (0·97, 1·64) | 2·04 (1·56, 2·68)*** | 1·68 (1·26, 2·23)*** | 0·87 (0·63, 1·22) | 1·43 (1·05, 1·94)* |
| **Student status** | No | REF | REF | REF | REF | REF |
|  | Yes, full time | 0·51 (0·35, 0·76)** | 0·58 (0·38, 0·89)* | 0·78 (0·51, 1·20) | 0·98 (0·63, 1·54) | 0·55 (0·36, 0·84)** |
|  | Yes, part time | 0·51 (0·31, 0·84) | 0·67 (0·39, 1·13) | 1·14 (0·69, 1·87) | 1·06 (0·60, 1·87) | 0·66 (0·38, 1·16) |
| **Making ends meet** | Difficult | REF | REF | REF | REF | REF |
|  | Neither easy nor difficult | 0·62 (0·47, 0·82)** | 0·49 (0·37, 0·65)*** | 0·37 (0·28, 0·50)*** | 0·49 (0·35, 0·67)*** | 0·91 (0·65, 1·27) |
|  | Easy | 0·50 (0·37, 0·67)** | 0·39 (0·29, 0·52)*** | 0·31 (0·23, 0·43)*** | 0·40 (0·28, 0·56)*** | 0·80 (0·57, 1·13) |
| **Education** | Low | REF | REF | REF | REF | REF |
|  | Medium | 0·73 (0·56, 0·95)* | 0·74 (0·56, 0·97)* | 0·65 (0·48, 0·88)** | 0·98 (0·70, 1·39) | 1·05 (0·77, 1·44) |
|  | High | 0·62 (0·49, 0·79)*** | 0·62 (0·48, 0·80)*** | 0·63 (0·48, 0·83)*** | 1·19 (0·88, 1·61) | 0·88 (0·67, 1·16) |
| Logistic regression models adjusted for sex, age, ethnicity, household composition, employment status, student status, ability, make ends meet, and educational level.  * P<0·05, **P<0·01, ***P<0·001 | | | | | | |
